# Integrative analysis of the PSMA family identifies PSMA6 as an adverse prognostic biomarker promoting bladder cancer cell proliferation

**DOI:** 10.7150/ijms.119034

**Published:** 2026-02-04

**Authors:** Zhengnan Huang, Xiangqian Cao, Yilin Yan, Huaxing Li, Bing Shen, Tengjiao Wang

**Affiliations:** 1 Department of Urology, Shanghai Ninth People's Hospital, Shanghai Jiaotong University School of Medicine, Shanghai 200011, China.; 2 Department of Urology, Shanghai Tenth People's Hospital, School of Medicine, Tongji University, Shanghai 200072, China.; 3 Urologic Cancer Institute, School of Medicine, Tongji University, Shanghai 200092, China.; 4 Tongji University Cancer Center, Shanghai Tenth People's Hospital, School of Medicine, Tongji University, Shanghai 200072, China.; 5 Shanghai Key Lab of Cell Engineering, Shanghai 200433, China.; 6 Department of Stem Cells and Regenerative Medicine, Translational Medicine Research Center, Naval Medical University, Shanghai 200433, China.

**Keywords:** bladder cancer, PSMA6, prognostic biomarker, proliferation, immune

## Abstract

**Background:**

Proteasome subunit alpha members (PSMAs) are reported to be involved in diverse biological processes, and mounting evidence indicates that PSMAs have been implicated in the carcinogenesis of various malignancies. Nevertheless, there is a scarcity of reports on the expression, prognostic significance, and potential functions of the PSMA family in bladder cancer (BLCA).

**Methods:**

We utilized the TCGA, GEO, TIMER, UALCAN, and HPA databases to evaluate the expression of PSMAs in BLCA. Survival analyses were performed using Kaplan-Meier methods. The validation of PSMA6 dysregulation in human BLCA samples encompassed western blotting and immunohistochemistry. For the enrichment of biological processes, we applied Gene Ontology (GO), Kyoto Encyclopedia of Genes and Genomes (KEGG), and Gene Set Enrichment Analyses (GSEA). Subsequent analyses involved the exploration of correlations between gene expression and Immune-related effects. In-depth investigations into the role of PSMA6 in BLCA were conducted through both *in vitro* and *in vivo* experiments.

**Results:**

We demonstrated that PSMA6 was upregulated among PSMAs in BLCA, and overexpression of PSMA6 was associated with unfavorable prognosis and tumor malignancy. Enrichment analyses disclosed the involvement of PSMA6 in immune-related activities within the tumor microenvironment. Furthermore, the expression of PSMA6 was closely correlated with tumor-infiltrating immune cells (TICs) and immune checkpoints (ICPs). Besides, we also revealed that BLCA patients with high PSMA6 expression would have better immunotherapy response. Functional studies demonstrated that PSMA6 knockdown suppressed BLCA cell proliferation *in vitro* and *in vivo*.

**Conclusions:**

Our findings suggested that PSMA6 might function as an unfavorable prognostic biomarker, fostering BLCA cell proliferation, while also potentially serving as a predictive indicator for the efficacy of immunotherapy in BLCA patients

## Introduction

Bladder cancer (BLCA) stands as the predominant malignant tumor within the urinary system, with its incidence demonstrating an upward trend over the years 1. According to the latest global statistics, new BLCA cases rank 12th among all cancers worldwide 2. The substantial morbidity and mortality associated with this disease impose a considerable burden globally. BLCA is classified into non-muscle-invasive bladder cancer (NMIBC) and muscle-invasive bladder cancer (MIBC) based on the depth of tumor invasion 3. Presently, surgical resection is considered the optimal treatment for NMIBC or MIBC patients meeting specific criteria. However, the notable challenge in the management of this disease lies in the high recurrence rate following surgery 4, 5. For recurrent or invasive BLCA, more systematic treatments including immunotherapy are especially needed to control disease progression and alleviate symptoms 6, 7. Therefore, aiming at the key targets of immunotherapy, exploring the biomarkers for early diagnosis, and finding better therapeutic targets are of great significance for enhancing the comprehensive treatment effect, reducing the recurrence rate of BLCA, and improving the prognosis of patients with this disease.

26S proteasome is a protein-degrading complex in eukaryotic cells that performs ATP-dependent intracellular protein degradation, this cellular structure functions through degradation and control of regulatory proteins 8. The 26S proteasome consists of a 20S core particle (CP) and two 19S regulatory particles (RPs). The 20S CP is in the middle and both ends are symmetrically covered by the 19S RP. The 20S CP barrel-shaped module, which performs proteolytic functions, is composed of two internal β ring-submits (PSMB) and two external α ring-submits (PSMA), which together form a hollow structure 9. PSMA family is the main functional subunit in 20S CP and consists of eight small subunits (PSMA1-8), which are encoded by gene PSMA1-8, respectively 10, 11. By specifically hydrolyzing target proteins through proteolysis, 26S proteasomes are involved in multiple biological activities, such as transcription factor activation, angiogenesis, cell division, and immune response. It has been reported that PSMA family could serve as valuable targets for anti-tumor therapy due to the diversity of its mediated biological processes and special roles in cancer cells 12-14. It has also been pointed out that the PSMA family members could act as potential targets in gastric cancer and cervical cancer 15, 16. These investigations suggested that members of the PSMA family held substantial significance across various cancer types. Nonetheless, there is a paucity of reports regarding the involvement of the PSMA family in BLCA.

In this study, diverse bioinformatics analysis approaches were employed to investigate the expression patterns and multifaceted clinical significance of PSMAs in BLCA. Our results unveiled a heightened expression of PSMA6 in BLCA, with its overexpression significantly correlated with an unfavorable prognosis and increased tumor malignancy. The attenuation of PSMA6 through silencing demonstrated a notable inhibition of BLCA cell proliferation both *in vitro* and *in vivo*. Furthermore, PSMA6 exhibited potential as a prospective predictive indicator, offering effective insights into forecasting the immunotherapy response among BLCA patients.

## Materials and Methods

### Data acquisition and pan-cancer analysis

The RNA-sequencing and clinical data were downloaded from the TCGA-BLCA (https://portal.gdc.cancer.gov/). Multiple datasets (GSE13507, GSE32548, GSE32894, GSE31684, GSE19915, GSE48075) downloaded from the Gene Expression Omnibus (GEO) were applied for further analysis (https://www.ncbi.nlm.nih.gov/geo/). TIMER (https://cistrome.shinyapps.io/timer/) and UALCAN (http://ualcan.path.uab.edu/) databases were also applied to examine the expression profile of PMSA family members in BLCA.

### HPA database for protein expression analysis

The Human Protein Atlas (HPA, http://www.proteinatlas.org/) serves as an extensive repository of information encompassing sequence data, pathology profiles, expression patterns, and distribution characteristics within diverse cancer tissues. Within the HPA database, an examination of the protein expression of the PSMA family was conducted, specifically in normal bladder epithelium and tissues afflicted with BLCA.

### Kaplan-Meier plot for survival analysis

The Kaplan-Meier plot was employed for the analysis of overall survival (OS) and disease-free survival (DFS) based on gene expression in the GEPIA2 database 17 (http://gepia2.cancer-pku.cn/#survival). The survival analysis involved the Mantel-Cox test, encompassing Cox proportional hazard ratios, 95% confidence intervals, and p-values.

### Functional enrichment analysis

PSMA6 high- and low-expression groups were divided according to RNA-seq results of TCGA-BLCA, with the median value as the cut-off. R package “edgeR” was used to identify differentially expressed genes (DEGs), |log2FC| > 1 and P < 0.05 were set as the screening criterion. GO, KEGG and GSEA analyses were used as the main enrichment analysis methods in our study. R package “clusterprofiler” was utilized to implement these analyses. The interactions between proteins were performed through GeneMANIA database (https://genemania.org/) 18.

### Tumor immune microenvironment study

CIBERSORT algorithm was utilized to analyze the proportion of 22 immune cells in BLCA samples of different expression subgroups. Immunophenoscores (IPS) of BLCA patients was retrieved from the Cancer Immunome Atlas (TCIA, https://tcia.at/home), the association of IPS and gene expression was investigated by the Wilcoxon rank-sum test.

### Cell lines and cell culture

Human BLCA cell lines (T24, 5637, J82, UM-UC-3, SCaBER, and RT4) originating from the Chinese Academy of Sciences Shanghai cell bank in China were acquired. T24, 5637, and RT4 cells were nurtured in RPMI-1640 (Gibco), while J82, UM-UC-3, and SCaBER cells were cultivated in Eagle's Minimum Essential Medium (Gibco). All culture media were supplemented with 10% fetal bovine serum (FBS) and penicillin/streptomycin. The cells were upheld in an environment saturated with 5% CO_2_ at 37 °C.

### Stable cell line construction

To achieve the knockdown of PSMA6, two short hairpin RNA (shRNA) plasmids targeting PSMA6 were engineered. These constructs were integrated into the lentiviral pLKO.1 backbone, incorporating puromycin resistance as a selection marker. The specific sequences of the shRNAs utilized were provided in Table S1.

### qRT-PCR

Cellular total RNA extraction was carried out utilizing TRIzol reagent (Invitrogen), followed by reverse transcription into complementary DNA (cDNA) using the HiScript III RT SuperMix Kit (Vazyme). Quantitative PCR reactions were conducted employing the ChamQ SYBR qPCR Master Mix (Vazyme). The primer sequences employed were provided in Table S1.

### Western blotting and immunohistochemistry (IHC)

Western blotting was carried out as previously described 19. Primary antibodies against PSMA6 (1:1000, 11573-1-AP, Proteintech) and Tubulin (1:1000, 11224-1-AP, Proteintech) were applied. The full uncropped western blotting images were presented in Figure S1. IHC staining of paraffin-embedded tissues with antibodies against PSMA6 (1:100, 11573-1-AP, Proteintech) and Ki67 (1:1000, ab15580, abcam) was conducted following the standard methods as previously reported 20.

### CCK-8

A cell seeding density of 2000 cells per well was employed in 96-well plates, followed by a subsequent incubation period. Subsequently, 90 μL of complete medium containing 10 μL of CCK-8 reagent (Dojindo) was introduced and allowed to incubate for 2 hours. The absorbance was then measured at 450 nm to assess cellular activity.

### Colony formation

A seeding density of 2000 cells per well was employed in 6-well plates. Following a 14-day incubation period, the cells were fixed using formaldehyde and subsequently stained with 0.2% crystal violet. The quantification of colony formation was then performed by counting the number of colonies.

### EdU

EdU labeling assays were executed utilizing the Cell-Light EdU Apollo488 *In vitro* Kit (C10310-3, RiboBio) in accordance with the manufacturer's guidelines. The EdU incorporation rate was quantified as the ratio of EdU-positive cells (GREEN) to the overall population of Hoechst33342-positive cells (BLUE).

### Xenograft tumor models

BALB/c nude mice, male and 5 weeks old, were randomly assigned to control and experimental groups (n=6/group). The dorsal flank of each mouse was injected with either 1×10^6 PSMA6-silenced BLCA cell lines or their respective control cell lines. Tumor volumes were measured at 4-day intervals and calculated using the formula: volume = (length × width^2^) /2. All procedures involving animals were subject to ethical review and approval by the Shanghai Tenth People's Hospital Medical Ethics Committee.

### Statistical analysis

In this study, statistical analyses were performed using R-Studio (Version 4.3.2) and GraphPad Prism (Version 9.0). For normally distributed variables, differences were assessed using the unpaired Student's *t*-test, whereas non-normally distributed variables were analyzed using the Wilcoxon rank-sum test. Correlation coefficients were calculated using Spearman's correlation analysis. P value < 0.05 was considered statistically significant. Statistical significance in the figures is denoted as follows: ns (not significant), *P < 0.05, **P < 0.01, ***P < 0.001.

## Results

### The expression of PSMA family members in pan-carcinoma and BLCA

**Figure 1:**
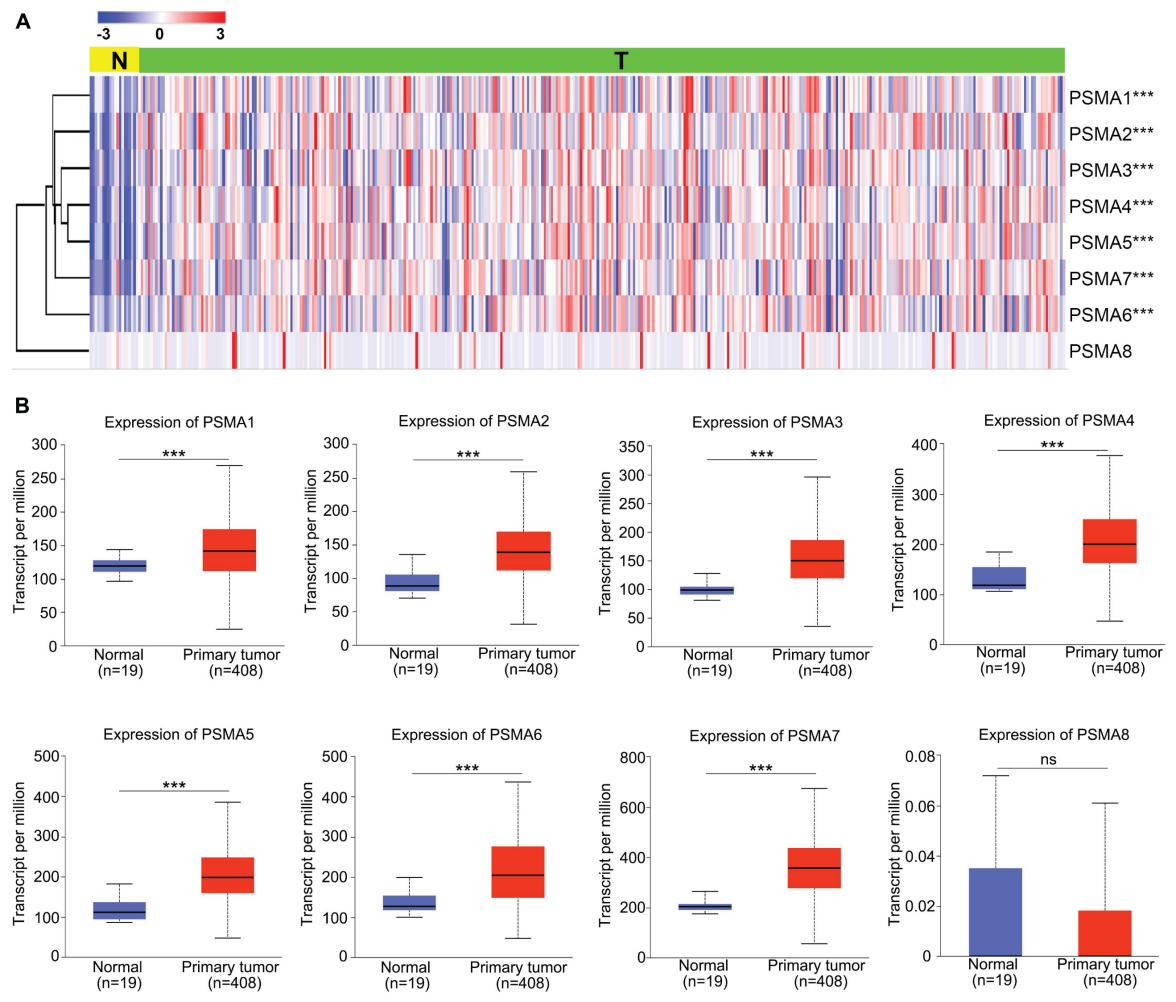
** Transcriptional level of the PSMAs in patients with BLCA. (A)** Heatmap of the PSMA family genes expression in TCGA-BLCA. **(B)** Relative differential expression of PSMA1-8 between BLCA tissues (T) and noncancerous counterparts (N), respectively. ns, not significant; ***P<0.001.

**Figure 2:**
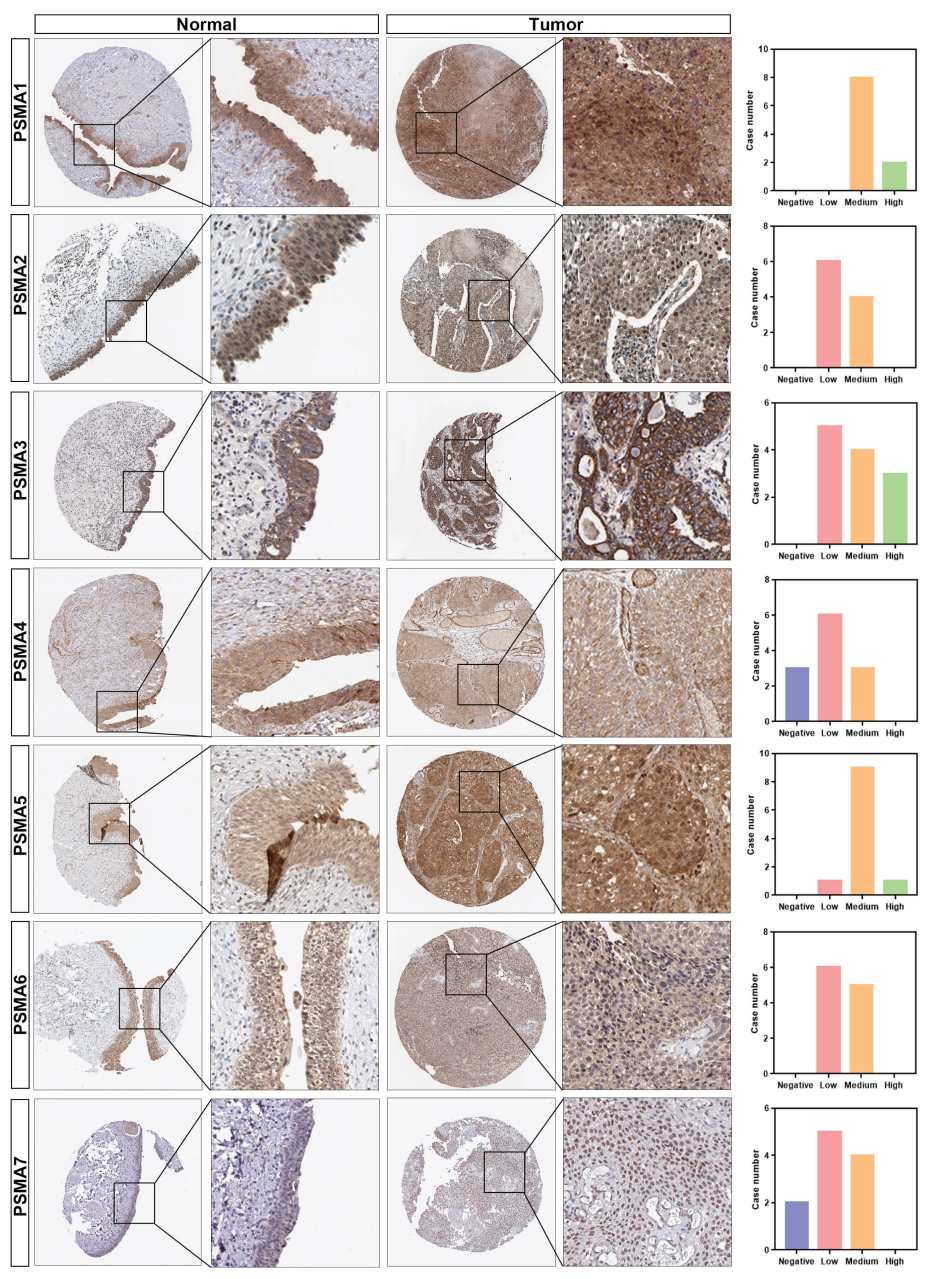
** Protein level of the PSMAs in patients with BLCA.** The protein level of PSMA1-7 in normal bladder tissue and bladder cancer was obtained from the HPA database. The local magnified figures were from the representative region, and the statistical figure was the statistics of staining results of all sections from the same antibody source.

To explore the potential significance of PSMAs in individuals with BLCA, the TIMER database was initially employed to assess the mRNA levels of PSMAs. As depicted in Figure S2, the expression patterns of PSMAs were presented across 20 types of cancers and their respective normal counterparts. Among these, we found that the expression of all PSMA family members except PSMA8 was significantly overexpressed in BLCA. Then, to further analyze the expression characteristics of PSMAs in BLCA, transcriptional level of the PSMAs was extracted from TCGA-BLCA cohort. The heatmap displayed the expression difference of 8 members of the PSMA family between normal and BLCA tissues (Figure 1A). Moreover, insights from the UALCAN database revealed a substantial elevation in the mRNA levels of PSMA1-7 in BLCA compared to normal tissues. Conversely, PSMA8 exhibited minimal expression in bladder tissue, with no discernible difference between normal and tumor tissues (Figure 1B). Overall, these findings underscored the noteworthy upregulation of PSMA1-7 expression in BLCA, while emphasizing the limited expression of PSMA8 in bladder tissue, implying a potentially insignificant role in BLCA. In addition, IHC data from the HPA database indicated distinct variations in the protein expression levels of PSMA family members between normal and BLCA tissues. Notably, the majority of PSMA family members exhibited elevated protein expression in BLCA compared to normal tissues (Figure 2).

### Prognostic value of PSMAs in BLCA patients

**Figure 3:**
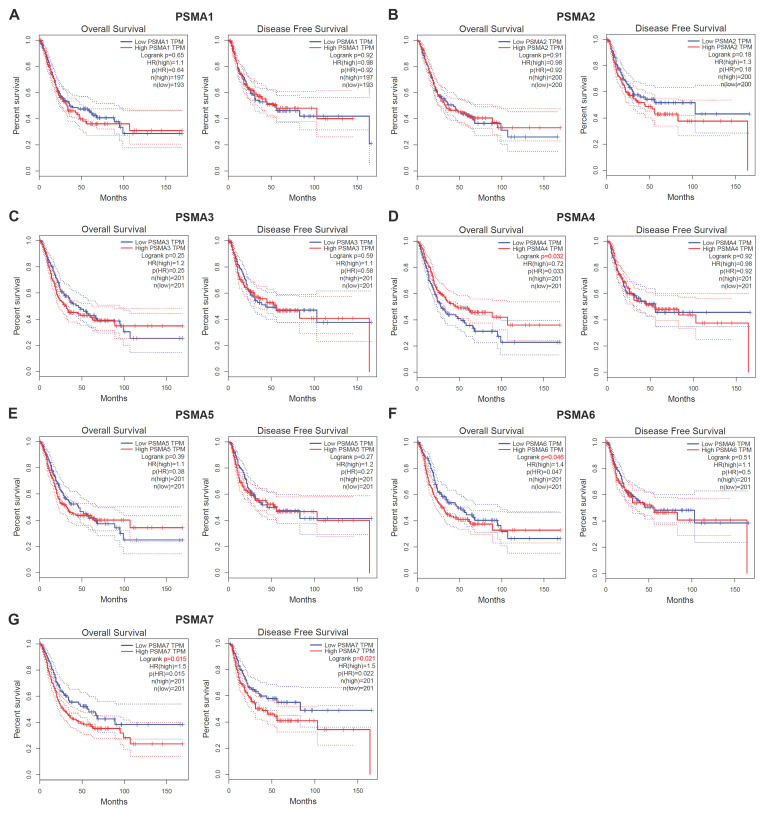
** Association of PSMA family genes expression and prognosis in patients with BLCA. (A-G)** The Overall Survival (OS) and Disease-Free Survival (DFS) analyses of PSMA1-7 in GEPIA2. The median expression level was used as the cut-off value.

To assess the prognostic significance of PSMAs in BLCA, Kaplan-Meier survival analysis was employed to investigate the association between PSMAs expression and prognosis. The results, presented in Figure 3, revealed noteworthy observations. Specifically, individuals with high expression levels of PSMA6 and PSMA7 in BLCA exhibited shorter overall survival compared to those with low expression (Figure 3F and G). Additionally, the high PSMA7 expression group demonstrated a lower disease-free survival rate compared to the low expression group (Figure 3G). Cumulatively, these findings suggested that elevated expression of PSMA6 and PSMA7 was indicative of an unfavorable prognosis in BLCA patients.

The upregulation of PSMA6 and PSMA7 in tumor tissues and their close association with poor prognosis suggest that both proteins may play critical roles in bladder cancer progression. Previous study using single-cell RNA sequencing have demonstrated that PSMA7 expression was significantly higher in MIBC compared to NMIBC. Furthermore, its expression level progressively increased with advancing clinical stages, demonstrating a strong correlation with tumor aggressiveness 21. Currently, the expression pattern and biological function of PSMA6 in bladder cancer remain poorly characterized. Therefore, this study focuses on PSMA6 to investigate its role in bladder cancer development.

### Overexpression of PSMA6 was correlated with tumor malignancy of BLCA

**Figure 4:**
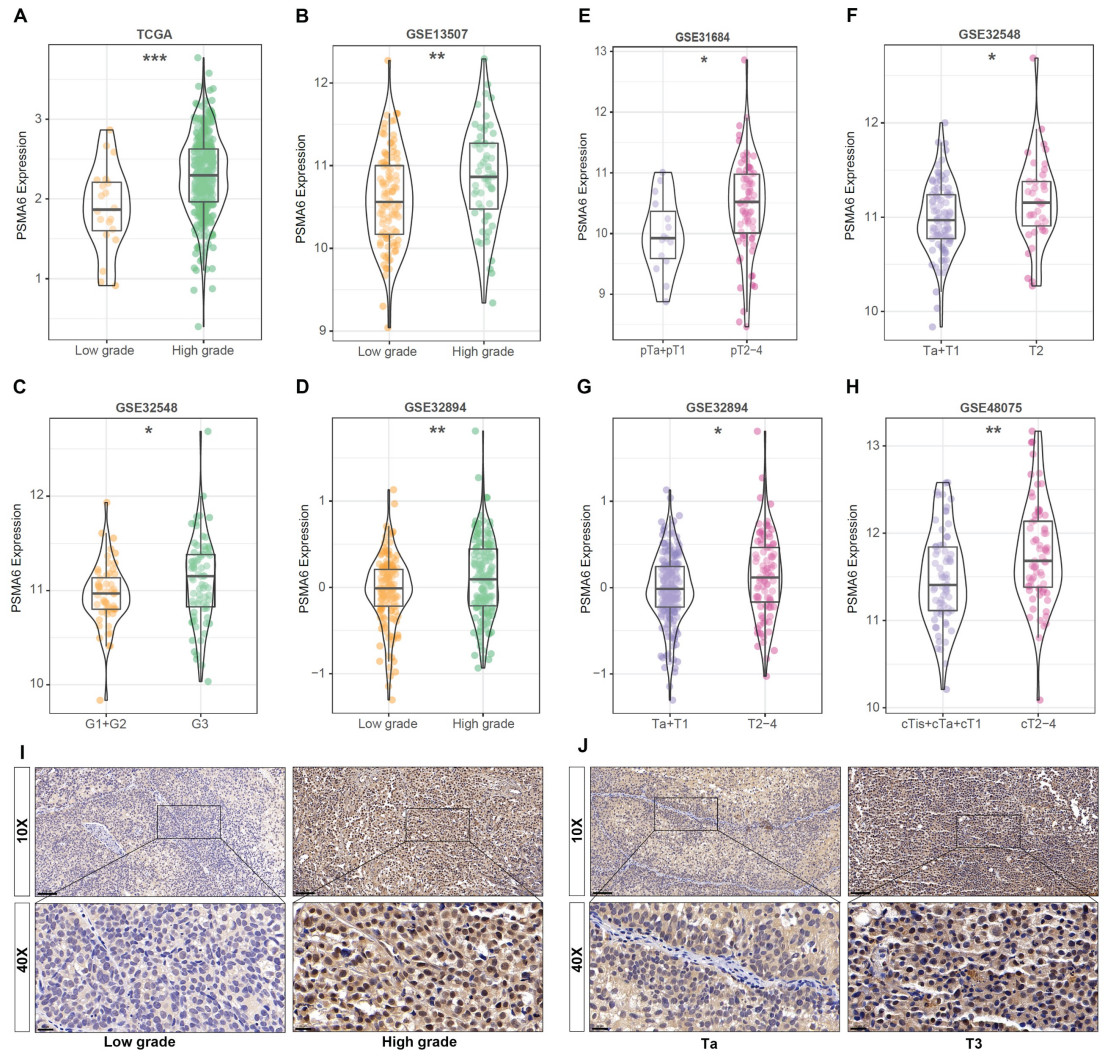
** Overexpression of PSMA6 correlated with tumor malignancy of BLCA. (A-D)** The association of the PSMA6 mRNA expression with tumor grade.** (E-H)** The association of the PSMA6 mRNA expression with pathological T stage.** (I, J)** Representative IHC images of the PSMA6 protein expression in indifferent pathological grade and stage. *P < 0.05; **P < 0.01; ***P < 0.001.

Concurrently, data derived from GEO databases indicated a notably higher expression of PSMA6 in high-grade BLCA patients compared to low-grade patients Figure 4A-D. Furthermore, a significant correlation was observed between PSMA6 expression and clinical stage (Figure 4E-H). Representative images of PSMA6 protein expression across different pathological grades and stages were depicted in the IHC data from our cohort (Figure 4I-J). These findings collectively substantiate the association of increased PSMA6 expression with the heightened malignancy of BLCA.

**Figure 5:**
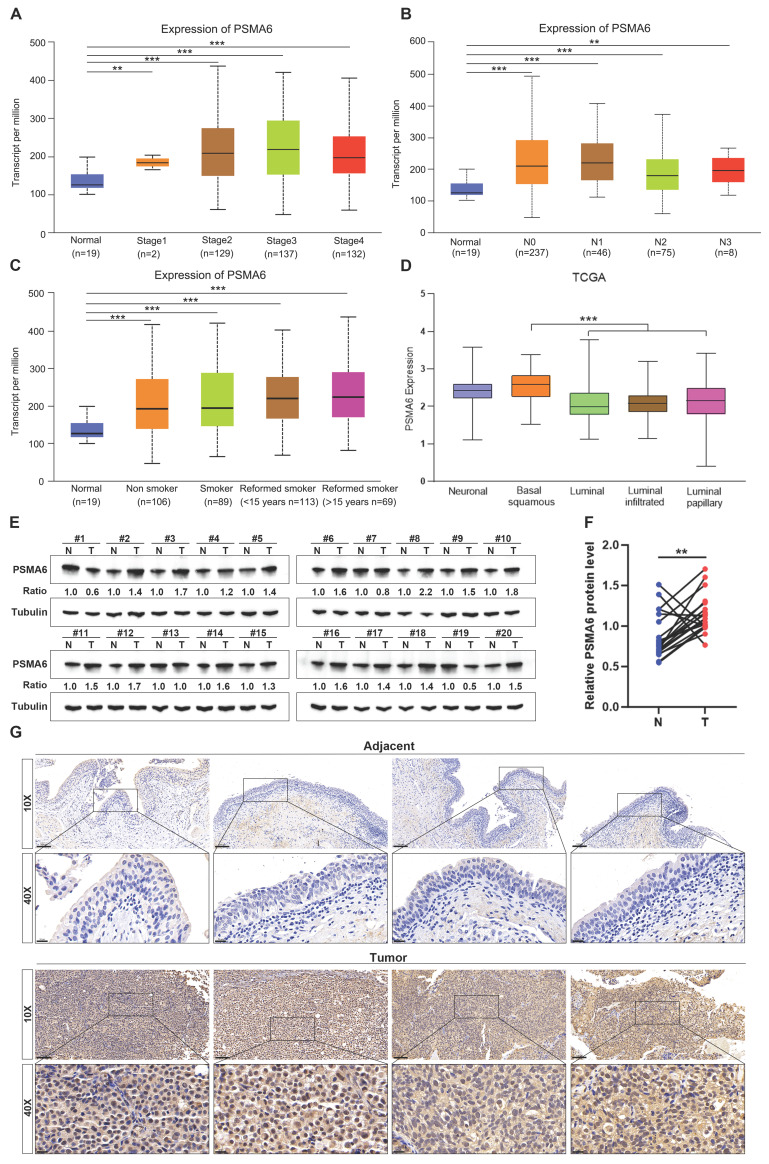
**PSMA6 was highly expressed in BLCA. (A-C)** Boxplots of PSMA6 relative expression based on cancer stage, lymph node metastasis and patient's smoking habit. **(D)** PSMA6 mRNA expression in five BLCA subtypes.** (E)** PSMA6 protein level in 20 paired BLCA tissues (T) and their adjacent normal urothelium tissues (N). **(G)** Representative IHC images of PSMA6 expression in noncancerous and BLCA tissues. Scale bar, 100 and 20 μm. **P < 0.01; ***P < 0.001.

Subsequently, the correlation between PSMA6 expression and clinical characteristics in BLCA patients was explored using the UALCAN database. Notably, the analysis of cancer stage, lymph node metastasis, and patients' smoking habits revealed a substantial elevation in PSMA6 expression in BLCA patients compared to normal controls (Figure 5A-C). The molecular subtypes of BLCA, categorized as Basal, Neuronal, and Luminal subtypes based on distinct genomic expression profiles 22, 23, exhibit varying prognostic characteristics and treatment sensitivities 24, 25. In this context, our investigation identified a particularly upregulated expression of PSMA6 in the basal squamous subtype, associated with a worse prognosis in BLCA (Figure 5D).

In summary, the aforementioned results collectively indicate an upregulation of PSMA6 in BLCA. The observed overexpression of PSMA6 is closely linked to tumor malignancy and prognostically signifies an unfavorable outcome, underscoring the potential pivotal role of PSMA6 in the progression of BLCA.

### PSMA6 was overexpressed in human BLCA tissues

To corroborate the findings derived from bioinformatic analysis, we scrutinized the expression of PSMA6 in human BLCA tissues. Initial western blotting assessments revealed a discernible upregulation at the protein level in 16 out of 20 pairs of the examined tissues (Figure 5E-F). Concurrently, IHC staining consistently illustrated notable expression of PSMA6 in the nuclei of BLCA cells, juxtaposed with weakly positive staining in normal urothelial cells (Figure 5G). These results provided additional confirmation that the expression of PSMA6 was indeed elevated in BLCA patients.

### Enrichment analysis of PSMA6 related genes in BLCA

**Figure 6:**
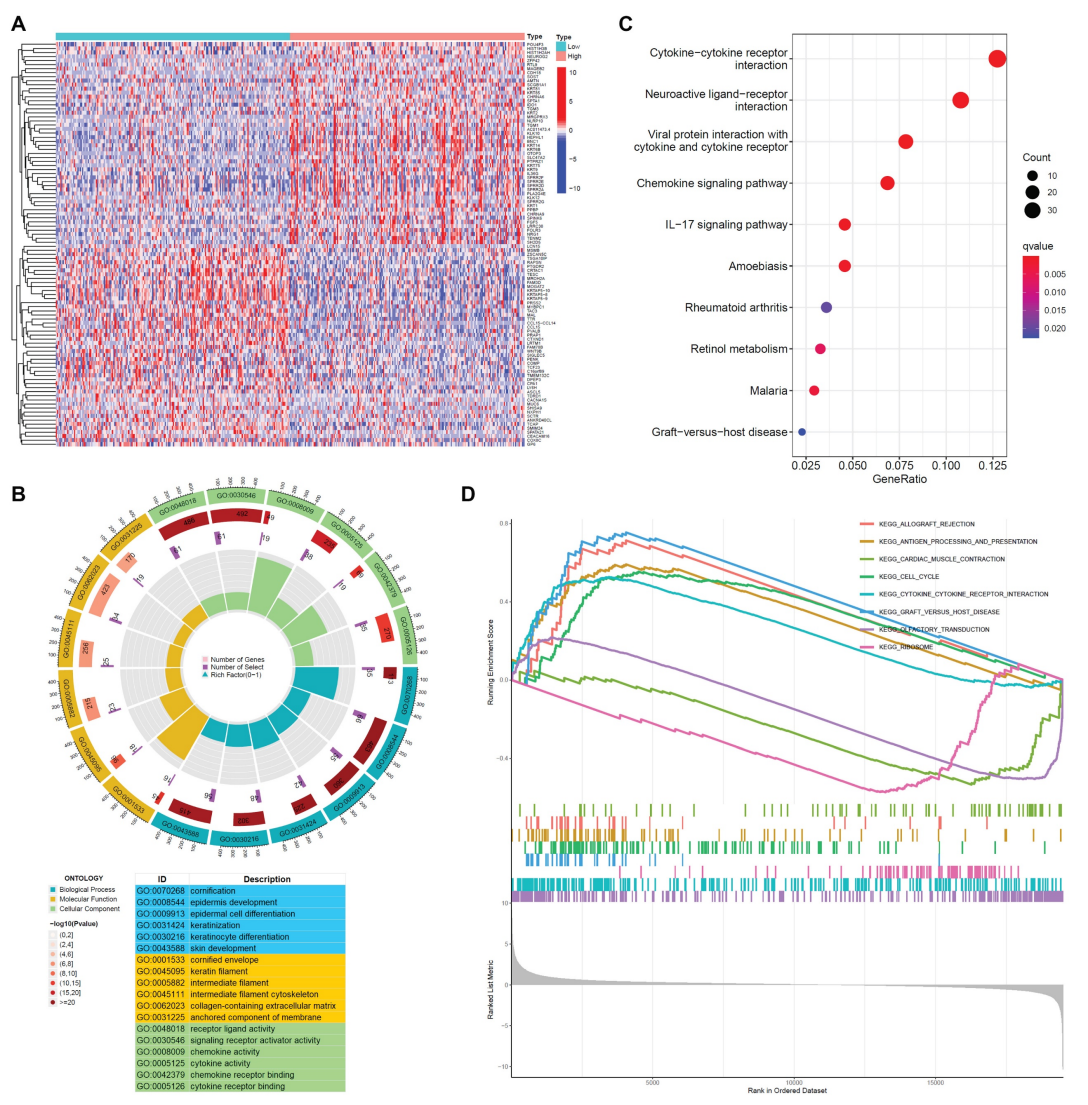
** Gene function enrichment analysis between PSMA6 high and low subgroups. (A)** Heatmap of DEGs between PSMA6 high-expression and low-expression groups.** (B)** GO enrichment analysis of DEGs.** (C)** KEGG enrichment analysis of DEGs.** (D)** GSEA results showing the top eight significant pathways.

To elucidate the plausible role of PSMA6 in BLCA, we performed enrichment analysis. Utilizing the RNA-seq data from TCGA-BLCA, samples were stratified into PSMA6 high- and low-expression groups based on the median value as the cut-off. The identification of DEGs was conducted using the R package “edgeR”, with |log2FC| > 1 and an adjusted P-value < 0.05 set as the screening criteria. The top 100 DEGs associated with PSMA6 were illustrated in Figure 6A. Subsequently, these DEGs underwent GO function and KEGG pathway enrichment analyses. Notably, GO functional analysis uncovered that DEGs of PSMA6 expression were primarily associated with cornification, cornified envelope, and cytokine activity (Figure 6B). KEGG analysis manifested that DEGs of PSMA6 expression were principally involved in immune-related activities, such as cytokine-cytokine receptor interaction (Figure 6C). Consistently, GSEA results also showed that immune-related pathways, especially antigen processing and presentation and cytokine-cytokine receptor interaction, were remarkably enriched (Figure 6D). Besides, we constructed the gene-gene interaction network for PSMA6 by using GeneMania. The results showed most of PSM family members such as PSMA3, PSMC6 and PSMC4, as well as STX16 and HDHD5 were closely correlated with PSMA6. Functional analysis suggested that these genes were mainly enriched in immune-related pathways (Figure S3). Together, above results indicated that PSMA6 might function via the involvement of regulation of tumor immune microenvironment. Thus, we further examined the association of PSMA6 with tumor immunity.

### PSMA6 was involved in tumor immunity in BLCA

**Figure 7:**
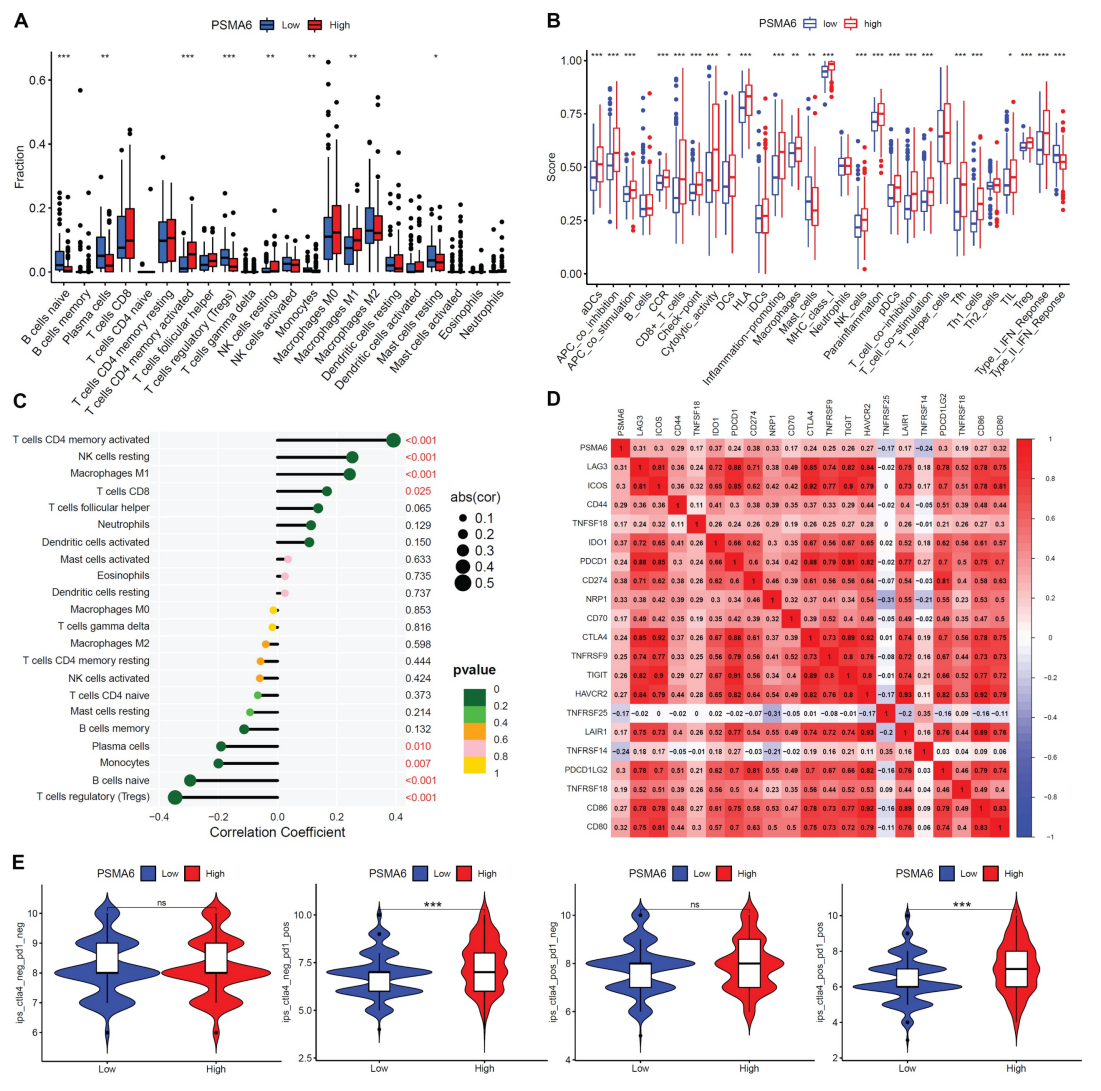
** PSMA6 was involved in tumor immunity in BLCA. (A)** Difference in the proportions of immune cell type in BLCA with low or high PSMA6 expression. **(B)** The association of PSMA6 expression with immune cell types and immune-related functions.** (C)** The correlation of PSMA6 expression with TICs.** (D)** The correlation of PSMA6 expression with immune checkpoint blocker.** (E)** The correlation of PSMA6 expression with IPS. ns, not significant; *P < 0.05; **P < 0.01; ***P < 0.001.

The CIBERSORT algorithm was first used to evaluate variations in TIC proportions between groups distinguished by high and low PSMA6 expression. The results revealed notable variances in activated CD4+ memory T cells, resting NK cells, M0 Macrophages, Tregs, naive B cells, Plasma cells, Monocytes, and resting Mast cells levels between the high and low PSMA6 expression groups (Figure 7A). Additionally, we discerned a close association between PSMA6 expression and the majority of immune cell categories as well as immune-related functionalities (Figure 7B). Further, we explored the correlation of PSMA6 with 22 kinds of immune cells, revealing that activated CD4+ memory T cells, resting NK cells, M1 Macrophages, CD8+ T cells had positive correlation with PSMA6, while Tregs had the strongest negative correlation (Figure 7C). We further investigate the correlations of PSMA6 and immune checkpoints and found that PSMA6 was positively correlated with multiple immune checkpoints, such as PD1 (PDCD1), PDL1 (CD274), CTLA4, and LAG3 (Figure 7D). Furthermore, the analytical outcomes of IPS in relation to PSMA6 expression revealed that individuals with elevated PSMA6 expression demonstrated a heightened IPS for anti-PD1 and anti-CTLA4 therapy (Figure 7E). Higher IPS was previously documented to be positively correlated with the enhanced immunogenicity 26, implying a better immunotherapy response. In summary, the above results highlighted that PSMA6 was associated with immune cell infiltration and could serve as a potential biomarker for effectively portending the effect of immunotherapy in BLCA.

### Knockdown of PSMA6 suppressed BLCA cell proliferation *in vitro*

**Figure 8:**
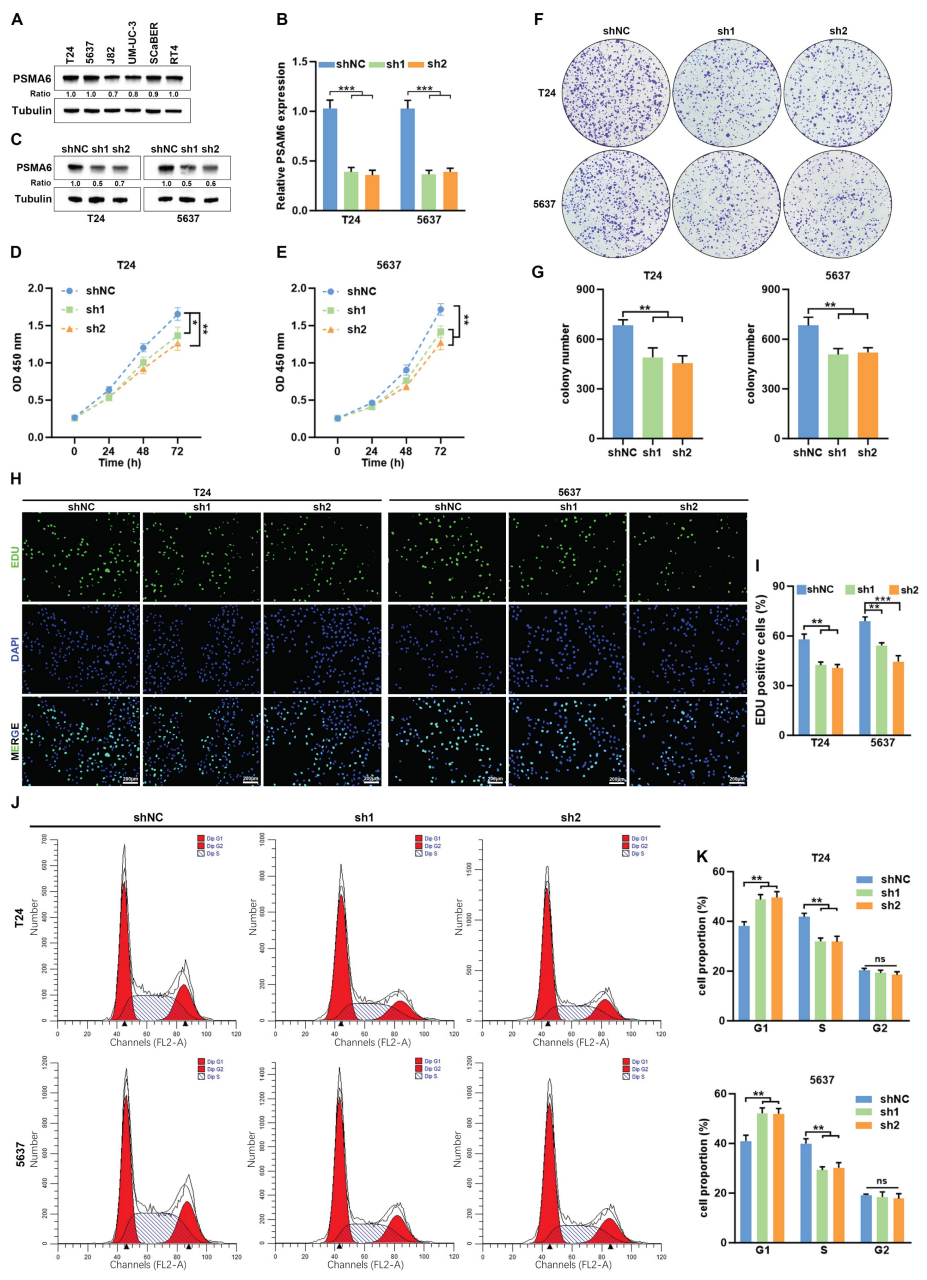
**Knockdown of PSMA6 suppressed BLCA cell proliferation *in vitro*. (A)** PSMA6 protein levels in multiple BLCA cell lines measured by western blotting. **(B, C)** Interference efficiency of two specific shRNAs (sh1 and sh2) to PSMA6 in BLCA cells was measured by qRT-PCR and western blotting. **(D-G)** Effects of PSMA6 knockdown on cell viability and colony formation. **(H, I)** Effects of PSMA6 knockdown on cell proliferation were determined by EdU. Scale bar, 200μm.** (J, K)** Effects of PSMA6 knockdown on cell cycle were determined by flow cytometry analysis. ns, not significant; *P < 0.05; **P < 0.01; ***P < 0.001.

In addition to its implication in immune-related processes, we observed a notable correlation between PSMA6 expression and a set of genes associated with the cell cycle (Figure 6D). This association suggests a potential involvement of PSMA6 in the regulation of proliferation and the cell cycle in BLCA. To validate this hypothesis, we initially investigated the impact of PSMA6 on cell proliferation. T24 and 5637 cells were selected for knockdown experiments due to their relatively higher endogenous PSMA6 expression among multiple BLCA cell lines (Figure 8A-C). As shown in Fig. 8D-G, knockdown of PSMA6 significantly suppressed cells viability and decreased colony numbers. Similarly, EdU assays also indicated inhibition of PSMA6 repressed the proliferation of BLCA cells (Figure 8H and I). Flow cytometry analysis revealed that PSMA6 depletion markedly blocked the cell cycle at the G1-S phase (Figure 8J and K). These findings revealed that PSMA6 promoted BLCA cell proliferation by regulating cell-cycle progression.

### Silencing of PSMA6 inhibited BLCA cell growth *in vivo*

**Figure 9:**
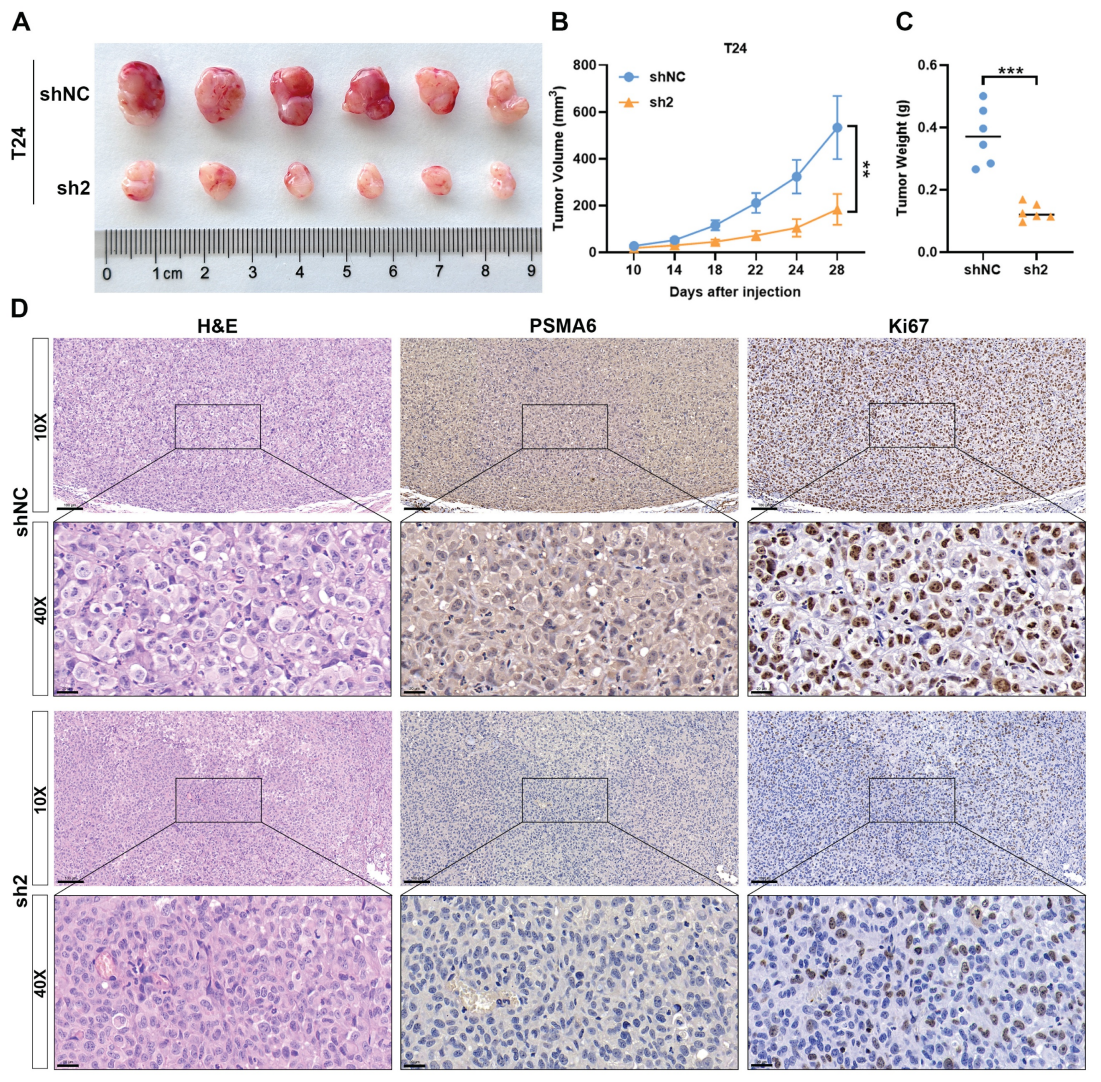
** Silencing of PSMA6 inhibited BLCA cell growth *in vivo*. (A)** Images of Xenografts from negative control (shNC) and PSMA6 silenced (sh2) groups (n = 6). **(B)** Tumor volume growth curves. **(C)** Average xenograft tumor weights. **(D)** Representative IHC images of PSMA6 and Ki67 protein expression in xenograft tumors. Scale bar, 100 and 20 μm. **P < 0.01; ***P < 0.001.

To delve deeper into the impact of PSMA6 on BLCA growth *in vivo*, xenograft tumor models were established. The inhibition of tumor growth was evident following PSMA6 knockdown, resulting in reduced tumor volume and weight compared to control groups (Figure 9A-C). IHC analysis of xenograft tissues further revealed a decrease in Ki67 protein levels in the sh-PSMA6 knockdown group compared to the control group (Figure 9D). Collectively, these findings signified that the suppression of PSMA6 impeded the growth of BLCA cells, both *in vitro* and *in vivo*.

## Discussion

As previously highlighted, BLCA stands as a global health concern with elevated mortality and morbidity rates 2. Timely diagnosis and effective management play pivotal roles in mitigating the burden and specific mortality associated with this cancer 27. Advancements in surgical therapy, chemotherapy, targeted therapy, and the widespread adoption of the early diagnosis and treatment paradigm have contributed to a partial improvement in the prognosis of BLCA over the years 27, 28. Nevertheless, the prognosis for individuals with advanced BLCA remains unfavorable, and the substantial recurrence rate poses a significant threat to the management of this disease 29, 30. From the perspective of cancer treatment, we hope to explore new targets for BLCA therapy and to better evaluate feasible treatment modalities based on the addition of disease diagnostic markers.

The ubiquitin-proteasome system is a functional system unique to eukaryotic cells, responsible for the degradation of more than 80% of proteins to maintain normal cell function 31. The role of the proteasome family in cancer has been well documented, and there is increasing evidence that the 26S and 20S proteasome were involved in tumor progression 32-34. The role of the proteasome subunit in cancer has also been gradually revealed. It was reported that the PSMD2 subunit played a significant role in regulating the proliferation of breast cancer cells by regulating the conversion of P21 and P27 through the ubiquitination process and deubiquitylation of USP14 35. Previous study also revealed that PSMA family had prognostic and predictive value in breast cancer and could serve a unique biomarker and potential prognostic indicator in breast cancer 11. Abnormal expression of PSMA family in tumors has been previously described. In Yang et al. study, PSMA1, LAP3, ANXA3, and maspin were identified as markers of colon cancer by proteomic analysis of antigen-induced immunogens in tumor tissue 36. PSMA2 was reported to be upregulated in advanced stage patients and could be a promising target for the therapy of colorectal cancer 37. Jesse et al. identified PSMA6 as an essential gene for its significant oncogenic role in pancreatic cancer cells using a genome-wide CRISPR screening test 38. According to a multiple myeloma study, the PSMA6 polymorphism (-8C>G) had a significant impact on the development and outcome of multiple myeloma, and patients with the PSMA6 CG+GG genotype had a higher probability of disease progression 39. Honma et al. demonstrated that PSMA7 was highly expressed in colorectal cancer and downregulation of PSMA7 effectively induced apoptosis in HT-29 cells 40. However, there has been no comprehensive and systematic study on PSMA family in BLCA.

In this study, we first explored the expression of 8 members in the PSMA family in different cancer types through pan-cancer analysis and discovered that PSMA1-7 showed a trend of overexpression in BLCA. We further verified this conclusion at RNA and protein levels respectively in TCGA-BLCA dataset and HPA online database. Through survival analysis, we determined that PSMA6 and PSMA7 had an impact on the prognosis of BLCA, and high expression of these two genes might be high-risk factors for poor prognosis. A thorough analysis of gene expression in conjunction with clinicopathological characteristics disclosed a significant correlation between the expression of PSMA6 and high pathological grade as well as advanced clinical stage. The aberrant overexpression of PSMA6 at the protein level was further validated in human BLCA samples by western blotting and immunohistochemical. Taking the above conclusions together, the dysregulation of PSMA6 expression in BLCA indicated that it might exert important role in the progression of BLCA.

There is a growing body of evidence pointing to PSMA6 have significant value in different types of disease. It was reported that inhibition of PSMA6 induced apoptosis and resulted in remarkably reduced cell viability, validating its significant oncogenic role in pancreatic cancer 38. In a diabetic nephropathy investigation, individuals with diabetic nephropathy exhibited diminished levels of PSMA6 protein compared to healthy controls. The reduction in PSMA6 was post-transcriptionally regulated by miRNA-4490 41. To illuminate the potential role of PSMA6 in BLCA, enrichment analysis was undertaken. The DEGs associated with PSMA6 were predominantly enriched in pathways related to the immune system. Furthermore, the expression of PSMA6 demonstrated a significant correlation with the regulation of immune cell infiltration and immune-related functions. Finally, we concluded that anti-PD1 and anti-CTLA4 therapy might achieve good clinical results in patients with high expression of PSMA6 through immunotherapy analysis. Furthermore, findings from both *in vitro* and *in vivo* experiments revealed that the knockdown of PSMA6 impeded the growth of BLCA cells, thereby underscoring the role of PSMA6 as an adverse biomarker in BLCA. However, the current study primarily establishes a correlative relationship between PSMA6 expression and immune microenvironment characteristics. Further investigations using *in vitro* and *in vivo* models are necessary to dissect the causal mechanisms-such as how PSMA6 might affect cytokine production, immune cell recruitment, or checkpoint molecule expression. This represents a limitation of the present work and will be a major focus of our future research.

Taken together, these findings suggested to us that future research can be attached to explore the specific mechanism of PSMA6 affecting immune response and cell proliferation, and further assess the possibility of PSMA6 as therapeutic targets and immunotherapy evaluation molecules.

## Conclusion

Overall, our findings revealed that PSMA6 might serve as an adverse prognostic biomarker promoting BLCA cell proliferation, as well as a prospective predictive indicator for immunotherapy effect of BLCA patients.

## Supplementary Material

Supplementary figures and table.

## Data Availability

All datasets used and analyzed during the current study are available from the TCGA, GEO, TIMER, UALCAN, HPA, GEPIA2, and GeneMANIA databases.
