# Supplementary material for: Integrative analysis of the PSMA family identifies PSMA6 as an adverse prognostic biomarker promoting bladder cancer cell proliferation
Source: Int J Med Sci. 2026 Feb 4;23(3):986–1001. doi: 10.7150/ijms.119034 (PMC12964584; doi:10.7150/ijms.119034)
Supplement: Supplementary file 1 — Supplementary figures and table. [file ijmsv23p0986s1.pdf]

Figure.5E

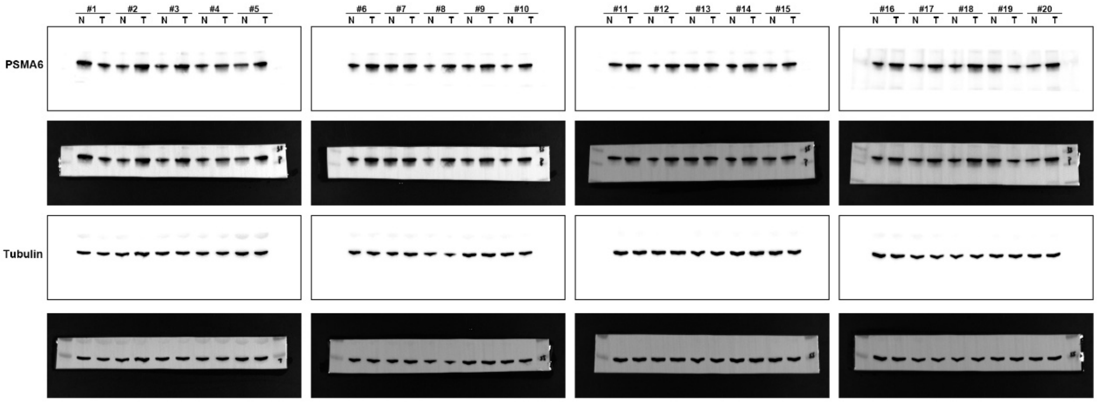

Figure.8A

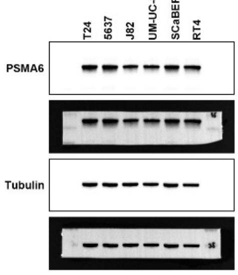

Figure.8C

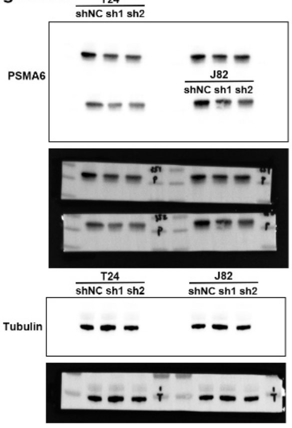

Supplementary Figure 1 The full uncropped western blotting images.

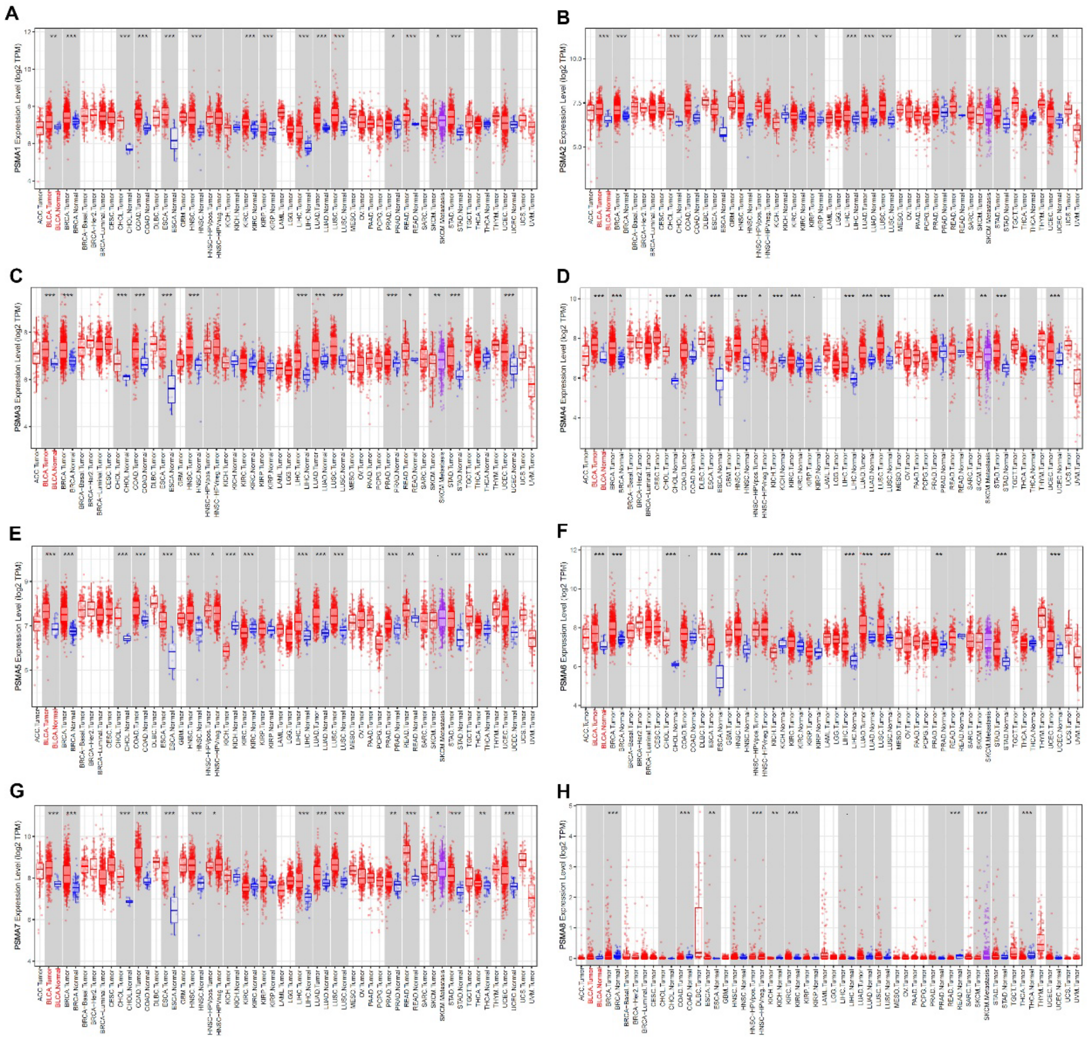

Supplementary Figure 2 Transcriptional level of the PSMAs in pan-carcinoma. **(A-H)** the mRNA level of PSMAs in 20 types of cancers and their normal counterparts.

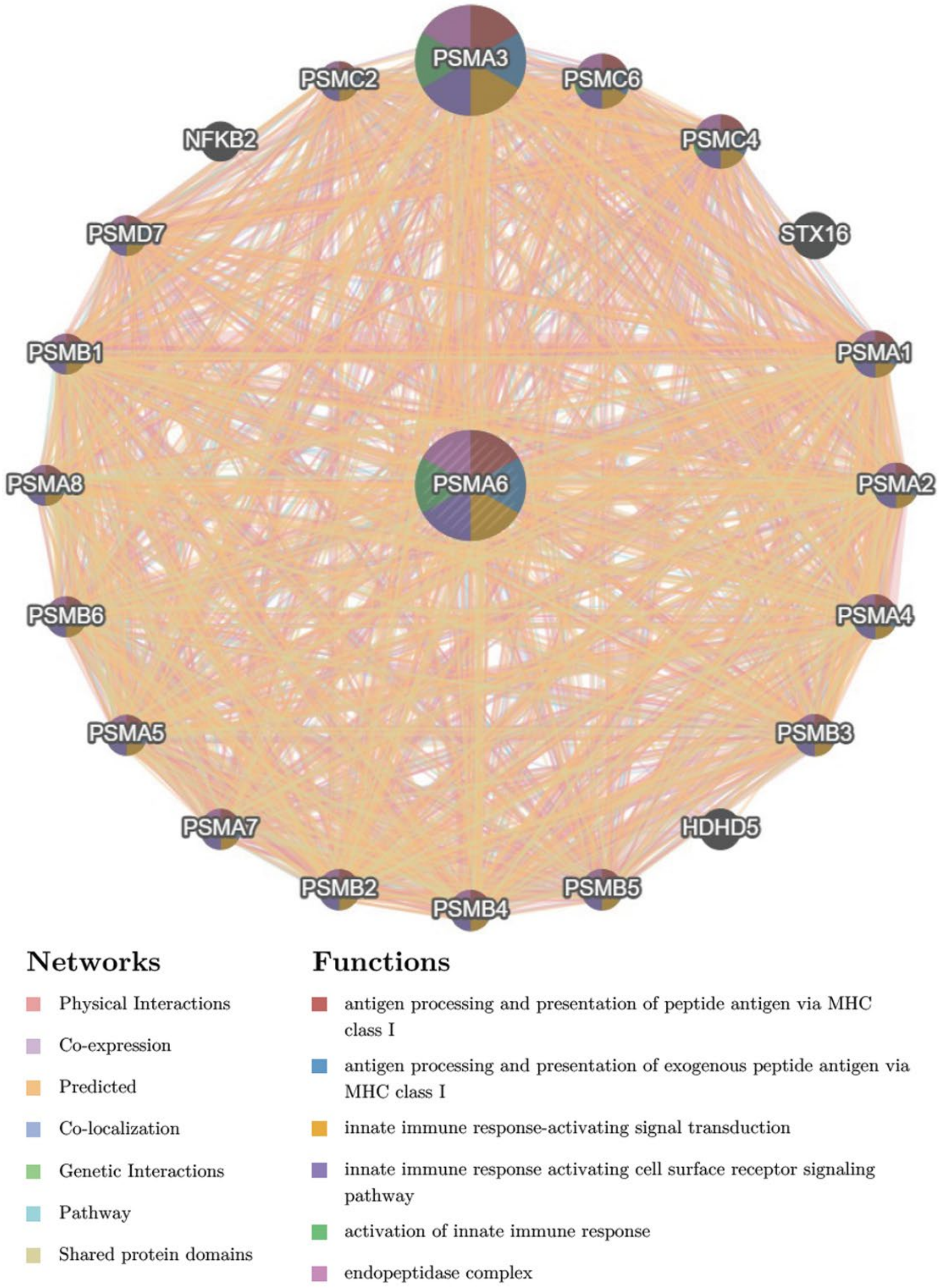

Supplementary Figure 3 Protein-protein interaction (PPI) networks of PSMA6 was visualized in GeneMANIA database.

Table S1 List for sequences of primer sets and shRNAs

	Direction	Sequences (5'-3')
**Primer sets for qRT-PCR**		
β-actin	Forward	CATGTACGTTGCTATCCAGGC
	Reverse	CTCCTTAATGTCACGCACGAT
PSMA6	Forward	GGTTTTGACCGCCACATTACC
	Reverse	GCCACCCTGGTTAATAGCCT
**shRNA sequences**		
shPSMA6-1	5' to 3'	ATGTCCCGTGGTTCCAGCGCC
shPSMA6-2	5' to 3'	CTCTACCAAGTAGAATATGCT
